# Is the Urban Child Health Advantage Declining in Malawi?: Evidence from Demographic and Health Surveys and Multiple Indicator Cluster Surveys

**DOI:** 10.1007/s11524-018-0270-6

**Published:** 2018-06-01

**Authors:** Edgar Arnold Lungu, Regien Biesma, Maureen Chirwa, Catherine Darker

**Affiliations:** 1 HIV Section, UNICEF Malawi, P.O Box 30375, Lilongwe, Malawi; 2 Department of Epidemiology and Public Health Medicine, Royal College of Surgeons in Ireland, Lower Mercer Street, Dublin 2, Ireland; 3 Prime Health Services and Consultancy, Area 47 Sector 4, Lilongwe, Malawi; 4 Department of Public Health & Primary Care, Trinity College Dublin, Tallaght Hospital, Dublin 24, Ireland

**Keywords:** Child health, Urban, Urban slum, Malawi, Urban advantage

## Abstract

In many developing countries including Malawi, health indicators are on average better in urban than in rural areas. This phenomenon has largely prompted Governments to prioritize rural areas in programs to improve access to health services. However, considerable evidence has emerged that some population groups in urban areas may be facing worse health than rural areas and that the urban advantage may be waning in some contexts. We used a descriptive study undertaking a comparative analysis of 13 child health indicators between urban and rural areas using seven data points provided by nationally representative population based surveys—the Malawi Demographic and Health Surveys and Multiple Indicator Cluster Surveys. Rate differences between urban and rural values for selected child health indicators were calculated to denote whether urban-rural differentials showed a trend of declining urban advantage in Malawi. The results show that all forms of child mortality have significantly declined between 1992 and 2015/2016 reflecting successes in child health interventions. Rural-urban comparisons, using rate differences, largely indicate a picture of the narrowing gap between urban and rural areas albeit the extent and pattern vary among child health indicators. Of the 13 child health indicators, eight (neonatal mortality, infant mortality, under-five mortality rates, stunting rate, proportion of children treated for diarrhea and fever, proportion of children sleeping under insecticide-treated nets, and children fully immunized at 12 months) show clear patterns of a declining urban advantage particularly up to 2014. However, U-5MR shows reversal to a significant urban advantage in 2015/2016, and slight increases in urban advantage are noted for infant mortality rate, underweight, full childhood immunization, and stunting rate in 2015/2016. Our findings suggest the need to rethink the policy viewpoint of a disadvantaged rural and much better-off urban in child health programming. Efforts should be dedicated towards addressing determinants of child health in both urban and rural areas.

## Background

Globally, there has been tremendous progress in reducing child mortality. A recent report by the United Nations Inter-agency Group for Child Mortality Estimation (UN-IGME) indicates that the total number of under-five child deaths dropped from 12.6 million in 1990 to 5.6 million in 2016, with the under-five child mortality rate having declined by 56%, from 93 to 41 deaths per 1000 live births [1]. Nonetheless, wide differentials exist in child mortality between and within countries. Reducing inequities and reaching the most vulnerable children (and their mothers) are important priorities to achieve the Sustainable Development Goals targets on ending preventable child deaths by 2030 [2].

Health indicators are on average better in urban than rural areas [3–6]. In Malawi, for example, under-five child mortality rate was 130 compared to 113 deaths per 1000 live births for rural and urban residents, respectively, in 2010 [7], and 77 and 60 deaths per 1000 live births for rural and urban residents in 2015 [8]. Other child health indicators generally reflect this trend of an urban advantage in many developing countries [3–6].

An analysis of under-five child mortality data in resource-poor settings noted a declining trend of child mortality in many countries, mostly with an urban advantage. Evidently, in the period between 1950 and 2000, under-five child mortality is said to have declined by 57% in both urban and rural areas [9]. However, over the same period, urban mortality patterns in Africa, Asia, and Latin America were reported to be 25% lower than rural mortality albeit acknowledging country variations in the urban-rural divide [9, 10].

Historically, prior to and in the early stages of industrialization, health indicators in urban areas of many countries in Europe were worse off than in rural areas. For example, evidence suggests that in the nineteenth century, infant mortality in urban areas in England and Wales were 2.2 times higher than in rural areas [9, 10]. With prevailing circumstances as these at the time, some authors have argued that the urban population could easily have been wiped out if it were not for high levels of in-migration [10].

The term *urban penalty* was prompted due to the phenomenon of worse health status of urban residents [11–13]. However, over the years, the public health revolution characterized by improved sanitation, access to safe water, vaccinations, and improved housing conditions led to improvements in urban health indicators to the extent that they became better than in rural areas. This health transition has gone on for a few decades, and the urban areas have enjoyed a health advantage over the rural areas leading to a phenomenon that has been termed the *urban advantage* [8, 14, 15].

Nonetheless, for about three decades now, since the urban health discourse has received some prominence in global health, some authors have argued that aggregate urban-rural comparisons suggesting an urban advantage are misleading considering that the urban population is not homogeneous [6, 12, 14–17]. Moreover, literature discourse has suggested that some population groups in urban areas, particularly those residing in urban slums, face similar levels of health disadvantage or in some cases actually face worse health outcomes than the rural areas [6, 12, 15–19]. In essence, poor health indicators in urban slums have been cited among reasons for the stagnating improvements in aggregate urban health indicators in some countries.

In recent decades, the world population has increasingly become urban based. The United Nations estimated that in 2016, about 55% of the world population were in urban settlements, and projected that this will increase to 60% by 2030. It is projected that most of the urban population growth will be occurring in least developed countries and that urban population will grow by 63% between 2015 and 2030 [20]. Four main reasons are cited as global determinants of increasing urbanization rates. These include (i) natural growth, whereby the existing urban population grows as a result of a high rate of natural increase (i.e., the difference between crude death rate and crude birth rate), (ii) internal rural-to-urban migration, (iii) international urban migration which relates to people moving from urban areas from one country to the other, and (iv) reclassification of urban boundaries encompassing formerly rural areas thereby increasing the urban population count by new geographical demarcations [21].

Malawi’s population of about 17.3 million in 2017 is predominantly rural based with only about 15% of the population residing in urban areas [22]. Different figures have been provided for Malawi’s urbanization rate from 4% [23] to as high as 6.2% which makes it among the highest in the world [24]. In Malawi, natural growth and rural to urban migration are arguably two main reasons attributable to the high urbanization rate. Evidently, there is a high total fertility rate of 4 among urban women in Malawi [7] and rural to urban migration accounts for 54% of total migration [25]. People migrate from rural to urban areas due to, *inter alia*, limited cultivable land in rural areas, lack of rural off-farm economic activities, environmental degradation resulting in inability to perform some of the conventional livelihood activities, and escaping rural poverty and the perception of a better life in the cities [26].

Poverty levels remain high with 74% of households in Malawi considering themselves poor [27]. In urban areas, this has led to emergence of urban slums characterized by inadequate access to clean water, sanitation, overcrowding, insecurity of housing tenure, and inadequate access to health and other social services [24, 28, 29], all of which are critical determinants of health. Indeed, the UN-HABITAT estimates that 61% of the urban population in Lilongwe, Malawi’s capital city, resides in slum conditions [24].

In view of the aforementioned evidence and context, the key question for public health in the urban setting, therefore, is whether there is any evidence of a declining urban advantage. This paper seeks to contribute to this area of urban health discourse, using under-five child health indicators as reported in five Demographic and Health Surveys (DHS) and two Multiple Indicator Cluster Surveys (MICS) in Malawi.

## Methods

### Study Design

We used a descriptive study undertaking a comparative analysis of 13 child health indicators between urban and rural areas using seven data points provided by nationally representative population-based surveys—the DHS and MICS. The use of under-five child health indicators for our focus is warranted on two premises.

Firstly, it is common consent that the health of children is sensitive to socioeconomic and environmental determinants such as economic development, general living conditions, social well-being, rates of illness, and the quality of the environment, all of which may reflect distinct differences between urban and rural geographic entities. Intuitively, using under-five child health indicators may closely reflect general health than other age groups. Indeed, infant mortality rate (IMR), for example, has long been regarded as a good proxy of population health albeit acknowledging arguments that contest this viewpoint [30]. However, some authors have argued that IMR is a safe indicator of population health and is not worse than some preferred measures in recent times, such as the Disability-Adjusted Life Expectancy (DALE) [30]. Secondly, under-five child health is a policy priority for the Ministry of Health in Malawi and many countries and hence essential to explore as a policy imperative in urban health discourse.

Rate changes over time for both urban and rural areas as independent geographical entities and rate differences for respective indicators between urban and rural areas are described to ascertain whether the urban advantage for child health is declining or widening or has remained static over the years in Malawi.

### Study Setting: Brief Country Profile

Our study uses national data for Malawi as the study setting. Malawi is a low-income country with an estimated per capita gross domestic product (GDP) of US$332 for 2016 [31]. Using the United Nations Development Programme’s (UNDP) Human Development Index (HDI) for 2016, Malawi is classified as a low human development country and is ranked 170 of the 187 countries [32]. Evidently, 50% of the population lives below the national poverty line of MK-101 (about US$0.3 according to prevailing exchange rates) per capita per day and 25% are considered to be ultra-poor (meaning they cannot afford to meet the minimum standard for recommended daily food requirement) [33].

Malawi’s epidemiological profile is characterized by a high burden of communicable diseases including malaria, acute respiratory infections (ARI), tuberculosis, and HIV and AIDS, albeit the burden of non-communicable diseases has recently been increasing. Pneumonia, diarrhea, HIV and AIDS, malaria and neonatal causes are the highest causes of morbidity and mortality for children under 5 years of age [34]. Despite a significant reduction in infant and under-five mortality, to an extent that Malawi achieved MDG 4 to reduce child mortality by two thirds between 1990 and 2015 [35], the rates are still high. The critical shortage of health system resources represents a challenge to effectively address the health problems of adults and particularly children. Evidently, per capita expenditure on health for 2012/2013 through 2014/2015 fiscal years was only at US$40 which falls far below the US$86 that the WHO Commission on Macroeconomics and Health recommends for delivery of basic health services for countries like Malawi [36]. Inadequate health workforce and inconsistent supply of essential medicines at the point of healthcare use also represent critical challenges [37].

### Data Sources

We used secondary data from five Malawi Demographic and Health Surveys (DHS) and two Multiple Indicator Cluster Surveys (MICS). The DHS are nationally representative household surveys usually conducted quadrennially by the ICF International in collaboration with governments in about 90 countries and provide data on a wide range of health and demographic indicators including mortality, sexual and reproductive health, HIV, health status and health seeking, and child nutrition [38]. The MICS surveys are conducted by various countries with support from UNICEF with an aim of providing internationally comparable data on the health status of children and women [39].

In Malawi, the DHS have been conducted in 1992, 2000, 2004, 2010, and 2015 [7, 8, 40–42], whereas the MICS have been conducted in 1995, 2006, and 2014 [43, 44]. The 1995 MICS report is not included because it did not provide information on some indicators used in this descriptive study. The 2014 MICS was used as an end-line survey to measure the country’s progress towards achieving the Millennium Development Goals. Both the DHS and MICS use nationally representative sample sizes and have used similar methodological approaches in measuring the indicators selected for this study; hence, their findings are highly comparable. In essence, the technical teams developing and supporting the DHS and MICS are in greater collaboration in recent times [45]. Granted that we used indicators as published in the DHS and MICS survey reports, the definitions of urban or rural areas as eligibility for our study were adopted from the two surveys. Both the DHS and MICS use robust data quality control measures throughout the data management process to the extent that their findings, including in both rural and urban settings, are highly regarded and utilized by researchers and policymakers.

### Selected Child Health Indicators for Analysis

Based on availability and comparability in all data sources, we selected and extracted 13 child health indicators, namely, neonatal mortality rate (NMR); infant mortality rate (IMR); under-five child mortality rate (U-5MR); stunting rate, prevalence of acute respiratory infections (ARI), fever, and diarrhea among children under 5 years of age; treatment seeking from a biomedical health provider for children with ARI, fever, and diarrhea; low birth weight; use of insecticide-treated nets (ITNs), and full immunization coverage.

### Data Extraction

Data for the urban and rural were extracted from respective DHS and MICS reports into a data abstraction matrix. The extraction of selected standard child health indicators applying the DHS and MICS definition and geographical entity of our interest—rural and urban—allowed for direct comparison of the indicator values and direct computation of the rate differences.

### Data Synthesis and Analysis

We calculated rate differences between urban and rural areas by subtracting the value of the child health indicator in a rural area from that reported for the urban area in the case of child health service utilization indicators (such as immunization coverage) and vice versa in the case of child morbidity and mortality indicators. This was intended to maintain the premise of an urban advantage for all indicators whereby a low value was subtracting from a larger value (thus expected low mortality in urban was subtracting from expected higher mortality in rural whereas expected low health service utilization in rural was subtracting from expected higher utilization levels in urban). We then plotted trends using rate differences to observe changes over time points of the population-based surveys, with a view of ascertaining whether the urban advantage was declining, increasing, or remaining constant.

## Results

### Trends in Child Mortality: Aggregate Improvements and Declining Urban Advantage

**Fig. 1:**
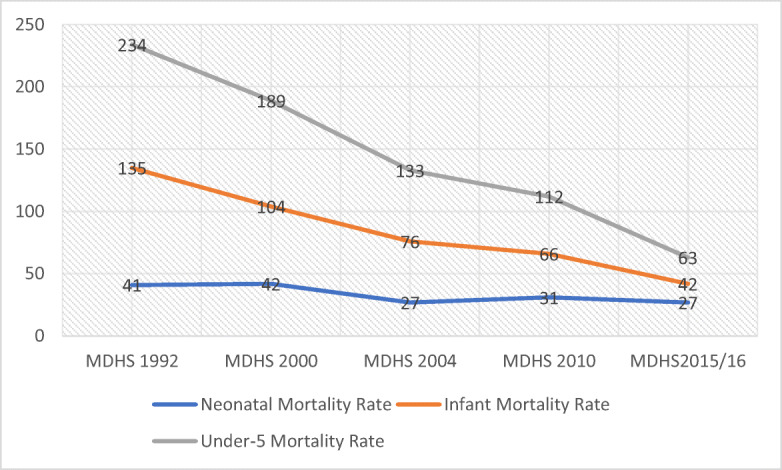
Trends in aggregate child mortality (NMR, IMR, and U-5MR) from 1992 to 2015/2016 in Malawi

The results show that there is an overall significant decline in neonatal mortality rate (NMR), infant mortality rate (IMR), and under-five mortality rate (U-5MR) from 1992 to 2015 in Malawi, as reflected in Fig. 1. The NMR declined from 41 to 27 deaths per 1000 live births; IMR declined from 135 to 42 and U-5MR from 234 to 63 deaths per 1000 live births.

**Fig. 2:**
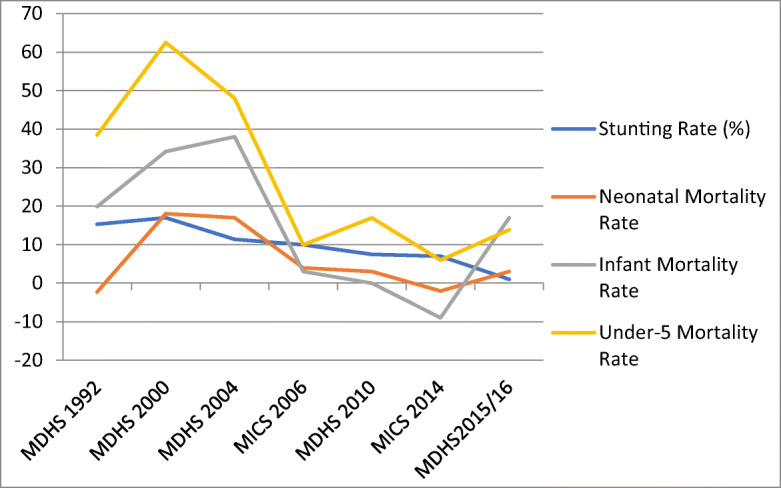
Rate differences in child mortality and stunting between urban and rural areas

Table 1 shows that whereas U-5MR has shown a consistent decline in both urban and rural geographical settings, for NMR and IMR, this pattern is only observed for rural areas where there is a consistent decline in all forms of child mortality. In urban areas, NMR and IMR show declining trends from 1992 DHS to the 2004 DHS but both increase in the 2006 MICS and 2010 DHS reports and for the NMR even in the 2014 MICS report. However, both NMR and IMR show a significant decline in the 2015/2016 DHS (Fig. 2).

**Table 1:** Child mortality (NMR, IMR, U-5MR) and stunting rates for urban and rural areas and rate differences between urban and rural levels

Child mortality indicators and stunting	Geographical area	DHS and MICS reports						
		MDHS 1992	MDHS 2000	MDHS 2004	MICS 2006	MDHS 2010	MICS 2014	MDHS 2015/2016
Neonatal mortality rate	Urban	50.9	29.8	22	30	31	31	26
	Rural	48.6	47.9	39	34	34	29	27
	RD	− 2.3	18.1	17	4	3	− 2	1
Infant mortality rate	Urban	118.1	82.5	60	70	73	61	44
	Rural	138	116.7	98	73	73	52	47
	RD	19.9	34.2	38	3	0	− 9	3
Under-5 mortality rate	Urban	205.4	147.9	116	113	113	80	60
	Rural	243.9	210.4	164	123	130	86	77
	RD	38.5	62.5	48	10	17	6	17
Stunting (%)	Urban	35	34.2	37.8	37.5	40.7	36.2	25
	Rural	50.3	51.2	49.2	47.5	48.2	43.2	38.9
	RD	15.3	17	11.4	10	7.5	7	13.9

Rate differences were calculated by subtracting urban values from rural values. This arrangement reflected the expected direction of health advantage

*RD* rate difference

Correspondingly, a comparison of urban–rural differentials shows that the rate difference between urban and rural child mortality rates has been declining up to 2014 and thereafter increasing in 2015/2016, as shown in a trend of rate differences of NMR, IMR, and U-5MR and a stunting rate in Fig. 1. This suggests a declining urban advantage relative to rural settings with regard to all forms of child mortality and stunting rates only up to 2014 and an increase reflected in the latest DHS but one which is not worse than that noticed in 2010 for NMR and U-5MR.

The urban advantage increased between 1992 and 2000 for stunting rate and IMR and U-5MR but afterwards showed a trend of a declining urban advantage reaching the same levels of IMR between rural and urban in 2010 (73 in both) and a reversal to a rural advantage in IMR in 2014 (52 vs 61; RD = − 9%). On the other hand, NMR started on a rural advantage in the 1992 DHS but reversed to an urban advantage in 2000, but thereafter the urban advantage has been declining to an extent that the rural setting retained its advantage as NMR was worse in urban than rural areas (as reflected by a rate difference below 0). However, a recent 2015/2016 DHS reflects wide differentials between urban and rural with an urban advantage in U-5MR as shown by a rate difference of 17 increasing from 6 in 2014 and similar to the rate difference (17) noted in 2010.

### Child Morbidity Indicators by Urban–Rural Place of Residence

**Fig. 3:**
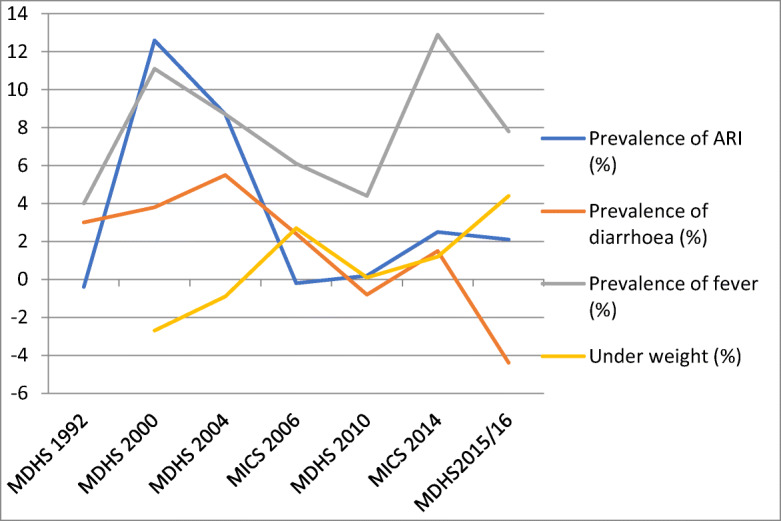
Rate differences in ARI, fever, diarrhea child morbidity, and underweight (prevalence) between urban and rural

A comparison of urban–rural differentials for child morbidity and nutrition (underweight) indicators show an unstable pattern. As shown in Table 2 and Fig. 3, the rate differences for prevalence of ARI show a slight rural advantage (less burden in rural compared to urban) in 1992 but shift to a wide urban advantage in 2000 before following a significant decline in urban advantage to the extent of an almost equal burden in 2006 (RD = − 0.2) and in 2010 (RD = 0.2) before moving to an urban advantage in 2014 and 2015/2016. Prevalence of fever indicates a slight urban advantage in 1992 but follows an increase in urban advantage in 2000 before following a declining urban advantage until 2014 when the urban advantage greatly increased to reaching the highest rate difference (RD = 13) of the review period. Both rate differences for prevalence of ARI and fever decline in the 2015/2016 DHS from the 2014 MICS levels.

**Table 2:** Child morbidity (ARI, fever, and diarrhea) and underweight for urban and rural areas and rate differences between urban and rural levels

Child morbidity indicators and underweight	Geographical area	DHS and MICS reports						
		MDHS 1992	MDHS 2000	MDHS 2004	MICS 2006	MDHS 2010	MICS 2014	MDHS 2015/2016
Prevalence of ARI (%)	Urban	14.9	15.7	11.3	8.7	6.6	5.6	3.6
	Rural	14.5	28.3	20	8.5	6.8	8.1	5.7
	RD	− 0.4	12.6	8.7	− 0.2	0.2	2.5	2.1
Prevalence of diarrhea (%)	Urban	19.3	14.3	17.5	22	18.2	22.7	25.5
	Rural	22.3	18.1	23	24.4	17.4	24.2	21.1
	RD	3	3.8	5.5	2.4	− 0.8	1.5	− 4.4
Prevalence of fever (%)	Urban	37	31.9	25.9	29.5	30.7	25.8	22.1
	Rural	41	43	34.6	35.6	35.1	38.7	29.9
	RD	4	11.1	8.7	6.1	4.4	12.9	7.8
Underweight (%)	Urban	NA	7.3	6.1	11.2	12.2	7.6	7.9
	Rural	NA	4.6	5.2	13.9	12.3	8.8	12.3
	RD	NA	− 2.7	− 0.9	2.7	0.1	1.2	4.4

Rate differences were calculated by subtracting urban values from rural values. This arrangement reflected the expected direction of health advantage. Data for children that were underweight was not available in the 1992 DHS

*RD* rate difference

Rate differences for prevalence of diarrhea have remained low across the surveys albeit showing a pattern of increase in urban advantage from 1992 to 2004 reports and like other child morbidity indicators showing a declining urban advantage through 2010 DHS which reflected a rural advantage followed by a slight urban advantage (rate difference of less than 2%) in the 2014 MICS report and a reversal to rural advantage (lesser diarrhea burden in rural by 4 percentage points) in 2015/2016 DHS. The pattern of urban–rural differentials with regard to children classified as underweight shows a rural advantage (greater burden of underweight children in urban areas) from 1992 through to 2004 when there was a reversal to an urban advantage (greater burden of underweight children in rural areas) in 2006, to equal burden and an urban advantage in 2014 MICS and 2015/2016. Except for underweight which has largely moved from a rural to an urban advantage, the rate differences for the rest of child morbidity indicators in 2015/2016 reflect lower levels than those of preceding 15 years (2000 DHS levels).

### Child Health Service Utilization Indicators by Urban–Rural Place of Residence

**Fig. 4:**
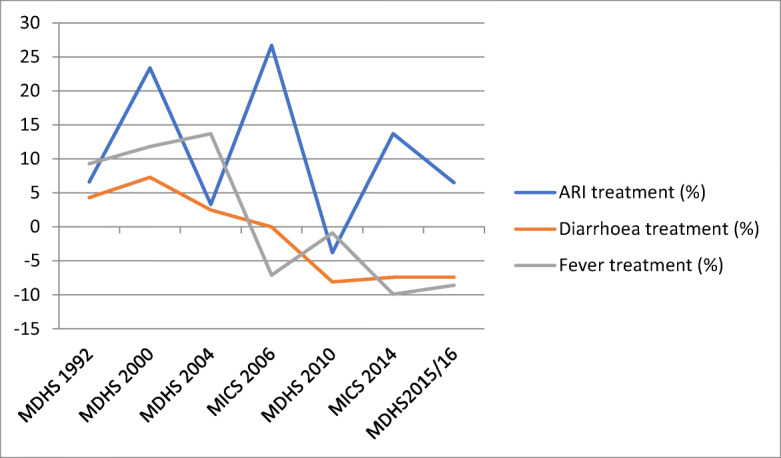
Rate differences in care-seeking for ARI, fever, and diarrhea between urban and rural areas

**Fig. 5:**
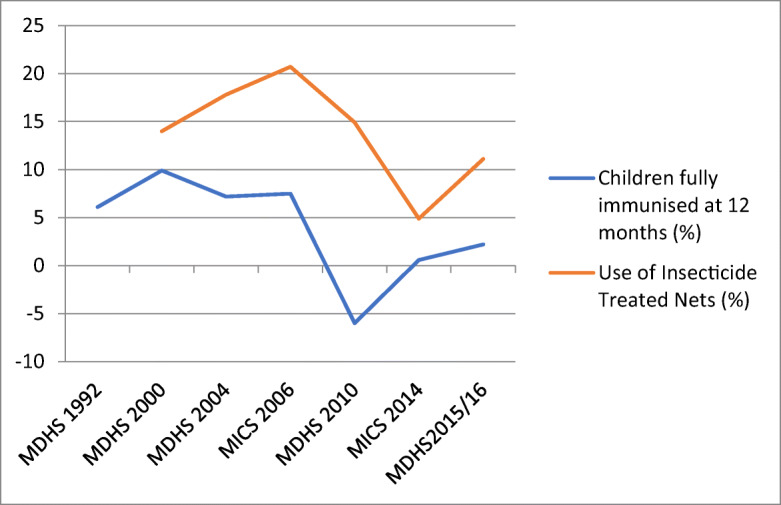
Rate differences in full immunization coverage and use of insecticide treated between urban and rural areas

Table 3 shows utilization rates and rate differences for child health services in urban and rural areas, and Figs. 4 and 5 show trends of urban–rural differentials with regard to utilization of essential child health interventions: biomedical treatment for ARI, fever, and diarrhea; use of insecticide-treated nets (ITNs), and full child immunization at 1 year of age. ARI treatment shows a rapidly fluctuating pattern of rate differences across the surveys. Utilization of diarrhea and fever treatment services clearly show a trend of declining urban advantage to an extent that rate differences are in the negative direction reflecting a rural advantage with regard to access to treatment for the two common childhood morbidities in the 2006, 2010, 2014, and 2015/2016 survey reports.

**Table 3:** Healthcare seeking for children with ARI, fever, and diarrhea for urban and rural areas and rate differences between urban and rural levels

Child health service utilization indicators	Geographical area	DHS and MICS reports						
		MDHS 1992	MDHS 2000	MDHS 2004	MICS 2006	MDHS 2010	MICS 2014	MDHS 2015/2016
ARI treatment (%)	Urban	54.8	48.3	22.6	74.5	67	32.6	83.5
	Rural	48.2	24.9	19.3	47.8	70.8	18.9	77
	RD	6.6	23.4	3.3	26.7	− 3.8	13.7	6.5
Diarrhea treatment (%)	Urban	49.3	34.9	38.7	NA	55.2	60.5	59.6
	Rural	45	27.6	36.2	NA	63.3	67.9	67
	RD	4.3	7.3	2.5	NA	− 8.1	− 7.4	− 7.4
Fever treatment (%)	Urban	54.5	45.8	42.6	20.2	42.6	65.8	59.1
	Rural	45.2	34	28.9	27.3	43.5	75.7	67.7
	RD	9.3	11.8	13.7	− 7.1	− 0.9	− 9.9	− 8.6
Children fully immunized at 12 months (%)	Urban	87.2	78.6	70.7	76.8	75.8	54.6	12.2
	Rural	81.1	68.7	63.5	69.3	81.8	54	10
	RD	6.1	9.9	7.2	7.5	− 6	0.6	2.2
Use of insecticide-treated nets (%)	Urban		19	30.2	42.3	85.9	72.8	52.4
	Rural		5	12.4	21.6	71	67.9	41.3
	RD		14	17.8	20.7	14.9	4.9	11.1

Rate differences were calculated by subtracting rural values from urban values. This arrangement reflected the expected direction of health advantage. For the MICS 2006, diarrhea treatment was classified differently (ORT and fluids) which was not directly comparable with other surveys thus indicated NA (not applicable)

*RD* rate difference

Data for use of ITNs was available from 2000 and reflects an urban advantage which continued an increasing pattern until 2006 when it rapidly declined up to 2014 although increased again in the 2015/2016 DHS. Full immunization coverage for children at 12 months also reflects a declining urban advantage over the years, moving to a rural advantage (RD = − 6) in 2010 albeit there is an almost equal utilization in 2014 (RD = 0.6) and a reversal to a slight urban advantage in 2015/2016 (RD = 2.2).

The results further show that across all health service indicators, even where there is evidence of an urban advantage, the rate differences remain low, typically below five percentage points for most of the recent survey reports with the exception of ARI treatment and use of ITN where rate differences exceed 15% in most survey reports.

## Discussion

This study sought to explore whether the urban advantage in child health indicators is declining in Malawi. The results show that all forms of child mortality have significantly declined between 1992 and 2015/2016 reflecting successes in child health interventions. Rural–urban comparisons, using rate differences, largely indicate a picture of the narrowing gap between the two geographical areas albeit the extent and pattern are different at the levels of child mortality, morbidity, and health service use.

Of the 13 child health indicators used in this study, eight (NMR, IMR, U-5MR, stunting rate, proportion of children treated for diarrhea and fever, proportion of children sleeping under ITN, and children fully immunized at 12 months) show clear patterns of a declining urban advantage particularly up to 2014. However, U-5MR shows a reversal to a significant urban advantage in 2015/2016, and slight increases in urban advantage are noticed for IMR, underweight, full childhood immunization, and stunting rate in 2015/2016.

Furthermore, of the eight, five (NMR, IMR, diarrhea treatment, fever treatment, and full immunization coverage) reach a point of reversal where one or more data points show a move from an urban to a rural advantage position. Four indicators (prevalence of fever, ARI, diarrhea, and treatment of ARI) have shown fluctuating trends with a declining urban advantage largely moving from 2000, 2004, to 2010 data points before another increase in 2014, except for ARI treatment which shows an unstable trend across all data points. Prevalence of underweight is the exception as it starts from a rural advantage when the data was first available in 2000 before a reversal to a slight urban advantage in 2006 MICS moving to almost equal levels in 2010 and a slight urban advantage in the 2015/2016 survey report.

A notably consistent decline in urban advantage with regard to all forms of childhood mortality is mainly due to a more rapid absolute decline in childhood mortality in rural areas. For health service-related indicators that show a declining urban advantage as aforementioned, it is seemingly due to two main reasons: (i) higher absolute increase in utilization of child health services in the rural areas, and (ii) lower absolute decrease in the rate of utilization of child health services in rural areas where the pattern in both urban and rural showed low utilization relative to the preceding survey. In some few cases, the narrowing gap between urban and rural is due to worsening of the indicator between one data point and another in the urban while there is an improvement between the same data points in the rural area. For example, while IMR increased in urban areas from 60 deaths per 1000 live births in 2004 to 70, 73, and 61 in the 2006, 2010, and 2014 reports, respectively, it largely reduced in rural areas over the same period from 98 to 73, 73, and 52 deaths per 1000 live births. Conversely, the increasing urban advantage typically noticed for some indicators in 2015/2016 is because of a faster improvement of respective child health indicators in urban areas and not necessarily worsening of indicators in rural areas.

Our findings suggest that for most indicators, a clear trend of declining urban advantage emerged for a large part of the years under review. This is consistent with other studies in Africa which have largely demonstrated the narrowing urban–rural gap with regard to child mortality and other determinants of childhood morbidity and mortality. Evidently, Garenne investigated trends in urban and rural mortality by reconstructing yearly mortality estimates from Welfare Monitoring Surveys (WMS) and DHS data from some sub-Saharan African countries which included Malawi in the periods from early 1970s to the late 1990s. The results, while generally affirming the declining trend in child mortality in both urban and rural settings, indicated that in some countries such as Burkina Faso, Rwanda, Senegal, Togo, and Uganda, mortality decline was faster in rural areas effectively narrowing the rural–urban gap. In Benin, urban mortality had stagnated while it continued declining in rural areas also reducing the rural–urban gap. In cases where the rural–urban gap had increased due to a faster mortality decline in urban areas such as Niger and Mozambique, the situation was reversed with data of the late 1990s [9]. Likewise, Murage et al. found that while there was an overall decline in childhood mortality in Kenya, urban–rural gaps in mortality narrowed and that mortality levels in urban slums showed a declining trend but remained high [19].

Furthermore, similar to our study, an analysis was conducted using DHS data to determine trends in urban–rural differentials of malnutrition among children aged 1 to 35 months for 15 sub-Saharan African countries. The results indicate a general decline in urban advantage in 8 of the 15 countries albeit with statistical significance in only two of these, no change in urban–rural differentials in four countries, and an increasing urban–rural gap in three of the countries. An increase in urban malnutrition was attributable to the declining urban advantage in some countries whereas a faster declining rate of urban malnutrition was responsible for the widening urban–rural gap in others [14].

On the basis of evidence from our descriptive study, it is clear that while there are some fluctuating patterns in some indicators, a trend of declining urban health advantage in so far as child health indicators are concerned in Malawi appears evident over the years. The underlying factors for this phenomenon are not obvious from the current study, but various hypotheses can be put forward for further interrogation in the context of Malawi but which have been highlighted in literature.

We postulate that the three salient factors proposed by Garenne and to some extent supported by other authors [14–19] as being responsible for the narrowing urban–rural gaps in health are applicable in Malawi. These factors, in aggregate terms, relate to determinants of urban health, and they include extreme urban poverty in some areas of the urban such as the urban slums often due to lack of state interventions; emerging diseases such as HIV and AIDS for which there is a greater disease burden in the urban than rural areas, especially in the pre-ART (anti-retroviral therapy, including prevention of mother-to-child transmission) era; and heightening risk of some diseases such as respiratory infections resulting from air and chemical pollution in cities.

Indeed, while Malawi is one of the least urbanized countries, its rate of urbanization is high and the majority (up to 61%) of people in Malawi’s capital city are said to be residing in slum conditions which embody urban poverty that manifest in limited access to improved water, appropriate sanitation, durable housing, sufficient living area, and insecurity of tenure [4]. The HIV factor is relevant granted that the HIV burden in Malawi shows geographic disparities and the urban HIV prevalence is almost twice as high (17.4%) as in rural areas (9%) [8]. Moreover, AIDS-related mortality accounted for about 13% and was among the top three causes of under-five mortality, and it can logically be argued that this affected the urban more than the rural at some point. The tremendous progress of the prevention of mother-to-child transmission (PMTCT) program in Malawi in recent years is however noted having registered a 71% reduction in mother-to-child transmission rate between 2009 and 2015 [46]. A successful PMTCT program in Malawi may explain greater survival of infants in the urban areas (which is disproportionately affected by HIV) and ultimately an increasing urban advantage in IMR as reflected in the 2015/2016 DHS report.

The heightened risk of respiratory infections due to air pollution cannot be backed by evidence from this study. In essence, the trend of ARI prevalence in the urban area seems to be that of a declining burden (see Table 2) albeit the cross-sectional nature of the national surveys used in this study is not the most appropriate to provide a true picture even when most surveys were undertaken over the same period of the year. Indeed, all the surveys ask for child morbidity in the 2 weeks preceding day of interview and would not be as precise in measuring a comprehensive morbidity burden as would a prospective study ascertaining incidence of ARI episodes over a given period. This notwithstanding, some underlying causes of child morbidity and mortality such as stunting rates have either stagnated, worsened, or dismally improved in urban areas over long periods and could play a critical role.

Our study has also shown that the urban advantage with regard to child health service use has been waning. In fact, when needed, some health service components such as diarrhea treatment, fever treatment, and childhood immunization have recently reversed from an urban to a rural advantage. In this regard, it would be argued that Malawi Ministry of Health policies of promoting access to health services for the rural population such as using Service Level Agreements, increasing health infrastructure, and undertaking community outreach clinics [37] may have yielded results. However, the findings also call into question the assumption that urban residents have adequate access to health services by virtue of geographical proximity relative to rural areas and that they ultimately have much better child health outcomes. Moreover, studies have demonstrated that access to health services transcends physical access [5, 47–49]. It is therefore imperative for the Ministry of Health in Malawi to rethink the policy premised on urban advantage pertaining to access to child healthcare services. Community health interventions such as child immunization and community case management of common childhood conditions like diarrhea could be considered especially in impoverished urban areas. Arguably, implementing an integrated Community Case Management (iCCM) component of IMCI in urban slums would be a form of differentiation of child healthcare delivery in the urban setting, effectively affording prompt access to essential child health interventions.

Some authors have argued that a stagnation of urban health levels, due to, among other reasons, the pervasive socioeconomic inequalities, has led to the narrowing of the urban–rural health gap [50]. Our study does not provide any evidence to this effect. The national surveys in this study do not report further socioeconomic quintile analysis by rural and urban geographical areas albeit it is possible to undertake a secondary analysis of their primary data. This was beyond the scope of this study but represents an area where further analysis is required granted the paucity of evidence of intra-urban child health inequities in Malawi and the effect of urban economic deprivation to overall urban health.

Could the declining urban advantage noted especially up to 2014 in this study merely be a phenomenon of the rural setting catching up with the urban? This is unlikely to be the case granted that the levels of child mortality and morbidity in urban areas also remain high and health service use is suboptimal, hence having more room for improvements at a rate similar to that in the rural areas or even better. Moreover, an increasing urban advantage in some child health indicators in the recent 2015/2016 DHS in the context of faster absolute improvements in urban relative to rural supports the assertion that there is still room for significant improvements in child health indicators in both settings.

We note some limitations to our study that should be taken into consideration when interpreting our results. We relied on already estimated values in DHS and MICS; hence, the limitations of these surveys such as recall bias and reporting bias should be borne in mind. The rigor in undertaking both DHS and MICS surveys used in this study and the fact that they are the most frequently used in shaping policy represent particular strengths.

## Conclusion

Using a total of 13 child health indicators reflecting impact level, morbidity, and health service use, the study has demonstrated that the urban child health advantage has largely declined in the past two and a half decades. The findings suggest the need to rethink the policy viewpoint of a disadvantaged rural and much better-off urban in child health programming. In particular, efforts should be dedicated towards addressing determinants of child health and this would arguably entail targeted interventions in urban slums for which arguments abide that they contribute to stagnation in some child health indicators and slow pace of improvements in other child health indicators.

Further research is warranted to validate some of our findings, particularly using yearly estimates and to ascertain levels of intra-urban inequities which arguably contribute to the declining urban advantage. It is worthwhile to note that the declining urban child health advantage itself is not a public health problem as rural progress is essentially a welcome development. Rather, it points to the need to pay as much attention to urban health to improve child health especially in the context of rapid urbanization and urbanization of poverty and other determinants of health. Reinvigoration and applying tenets of the urban health movements such as Healthy Cities Initiatives [51] and Participatory Slum Upgrading Programs (PSUP) [52] are needed in Malawi now as the country continues to urbanize at a fast pace and provision of quality health and other social services remains a challenge. Additionally, the state of population health in peri-urban areas (outskirts of urban geographical areas) may require further research granted its geographical position which is in the midst of rural and urban classification and its context arguably poses unique determinants of health. The overarching principle of leaving no one behind promulgated by the Sustainable Development Goals warrants renewed commitment to universal health coverage.
